# Bidirectional Interaction Between the Brain and Bone in Traumatic Brain Injury

**DOI:** 10.1002/advs.202503149

**Published:** 2025-07-14

**Authors:** Wei Zhang, Jun Zou, Lingli Zhang

**Affiliations:** ^1^ School of Exercise and Health Shanghai University of Sport Shanghai 200438 China; ^2^ College of Athletic Performance Shanghai University of Sport Shanghai 200438 China

**Keywords:** bone formation, bone marrow mesenchymal stromal cells, brain–bone axis, fracture healing, osteoporosis, traumatic brain injury

## Abstract

Traumatic brain injury (TBI), which refers to damage caused by external forces to the brain, significantly affects systemic organs and tissues, especially bone homeostasis. An increasing number of studies have revealed bidirectional crosstalk between the brain and bone, and the interactions between these systems in the context of TBI remain unclear. Here, existing research on the relationship between the brain and bone is summarized to explore their interactions and underlying mechanisms in TBI. Clinical studies indicate that long‐term loss of bone mass and increased risk of osteoporosis occur in patients after TBI. Interestingly, the rate of bone healing is accelerated when patients with TBI also suffer from fractures, which then worsens the prognosis of TBI. The bidirectional effects and underlying mechanisms that connect TBI and bone through neurohormones, neuropeptides, neurotransmitters, and mechanical factors are reviewed. The promising applications of bone marrow mesenchymal stromal cells, their derived extracellular vesicles, and bone‐derived factors for TBI recovery are also elucidated. Strategies to prevent osteoporosis management and potential mechanisms to accelerate fracture healing after TBI are proposed based on the brain–bone axis, and results are expected to translate into a clinical scenario for TBI and bone disease.

## Graphical abstract

Traumatic brain injury (TBI) disrupts the blood–brain barrier and activates neuroimmune responses, causing metabolic disturbances and long‐term bone mass loss. Concurrent fractures accelerate healing and enhance osteogenesis but disrupt regulatory mechanisms, leading to altered bone dynamics and exacerbating neuroinflammation, complicating recovery from TBI. Understanding these interconnected processes is crucial for developing therapeutic strategies to mitigate bone and neurological complications in TBI.

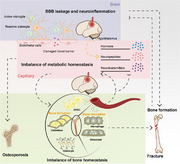

## 1 Introduction

Traumatic brain injury (TBI) is the disruption of brain function and brain lesion caused by external physical injury and is a major public health problem. TBI is caused by road traffic accidents, falls, violence, and other causes.^[^ ^1^ ^]^ Approximately 50 million new cases of TBI occur annually worldwide, with children and the elderly identified as high‐risk populations.^[^ ^2^ ^]^ The estimated annual mortality attributable to TBI ranges from 250 000 to 500 000 individuals. In China, the population mortality rate from TBI is ≈13 per 100 000, similar to values reported from other countries.^[^ ^3^ ^]^ Focal and diffuse injuries are most commonly observed in TBI. Focal injuries are generally caused by direct or indirect impact and present as contusions and subdural hematomas. Diffuse injuries are usually caused by rapid acceleration and deceleration of the head and manifest as extensive lacerations of axons and small blood vessels from shear forces.^[^ ^4^ ^]^ Refractory intracranial hypertension caused by massive cerebral contusion, intracerebral or subdural hematoma, and cerebral edema in patients with severe TBI is associated with poor clinical outcomes.^[^ ^5^ ^]^ TBI occurring in later life also increases the risk of dementia and Parkinson's disease.^[^ ^6^, ^7^ ^]^

Homeostasis of bone metabolism is a dynamic physiological process regulated by central signals from the brain.^[^ ^8^, ^9^, ^10^ ^]^ Large‐scale genetic correlation studies show that brain structure correlates with bone mineral density (BMD) in humans.^[^ ^11^ ^]^ Peripheral information is integrated by hypothalamic neurons and glial cells and converted into physiological outputs involved in the regulation of metabolism and skeletal homeostasis.^[^ ^12^ ^]^ The regulation of body metabolism and bone mineral density by hypothalamic neurons was glycopeptide‐dependent.^[^ ^13^ ^]^ A GABAergic projection from the subfornical organ (SFO) to the paraventricular nucleus (PVN) modulates parathyroid hormone (PTH) and bone mass.^[^ ^14^ ^]^ In addition, orexin produced in the hypothalamus is a critical rheostat of skeletal homeostasis,^[^ ^15^ ^]^ while leptin signaling in the hypothalamus regulates bone formation via the sympathetic nervous system.^[^ ^16^ ^]^ Bone metabolism and homeostasis are regulated not only indirectly by the central nervous system (CNS) but also directly by the peripheral nervous system (PNS). The expression of nicotinic and muscarinic acetylcholine receptors on osteoblasts, osteocytes, and osteoclasts indicates that these cells are directly regulated by the parasympathetic nervous system.^[^ ^17^ ^]^ Beta‐adrenergic receptors are the primary adrenergic receptors mediating sympathetic effects on bone.^[^ ^18^ ^]^ The detection of the expression of calcitonin gene‐related peptide (CGRP) and substance P in bone, as well as the expression of neuropeptide receptors in osteoblasts and osteoclasts, suggests the presence of direct sensory fiber innervation in bone.^[^ ^19^ ^]^ Bone homeostasis reveals specific regulatory mechanisms in different environments or diseases. The bed nucleus of the stria terminalis (BNST)–ventromedial hypothalamus (VMH)–nucleus tractus solitarius (NTS) neural circuitry under extreme environmental stimuli regulates stress‐induced bone loss through the peripheral sympathetic system.^[^ ^20^ ^]^ In pathological conditions, such as Alzheimer's disease (AD), the interplay between the brain and bone becomes evident because patients with AD often exhibit symptoms of brain degeneration accompanied by decreased bone mass.^[^ ^21^ ^]^ This finding underscores the significance of CNS regulation in physiological and pathological contexts regarding skeletal health.

**Figure 1:**
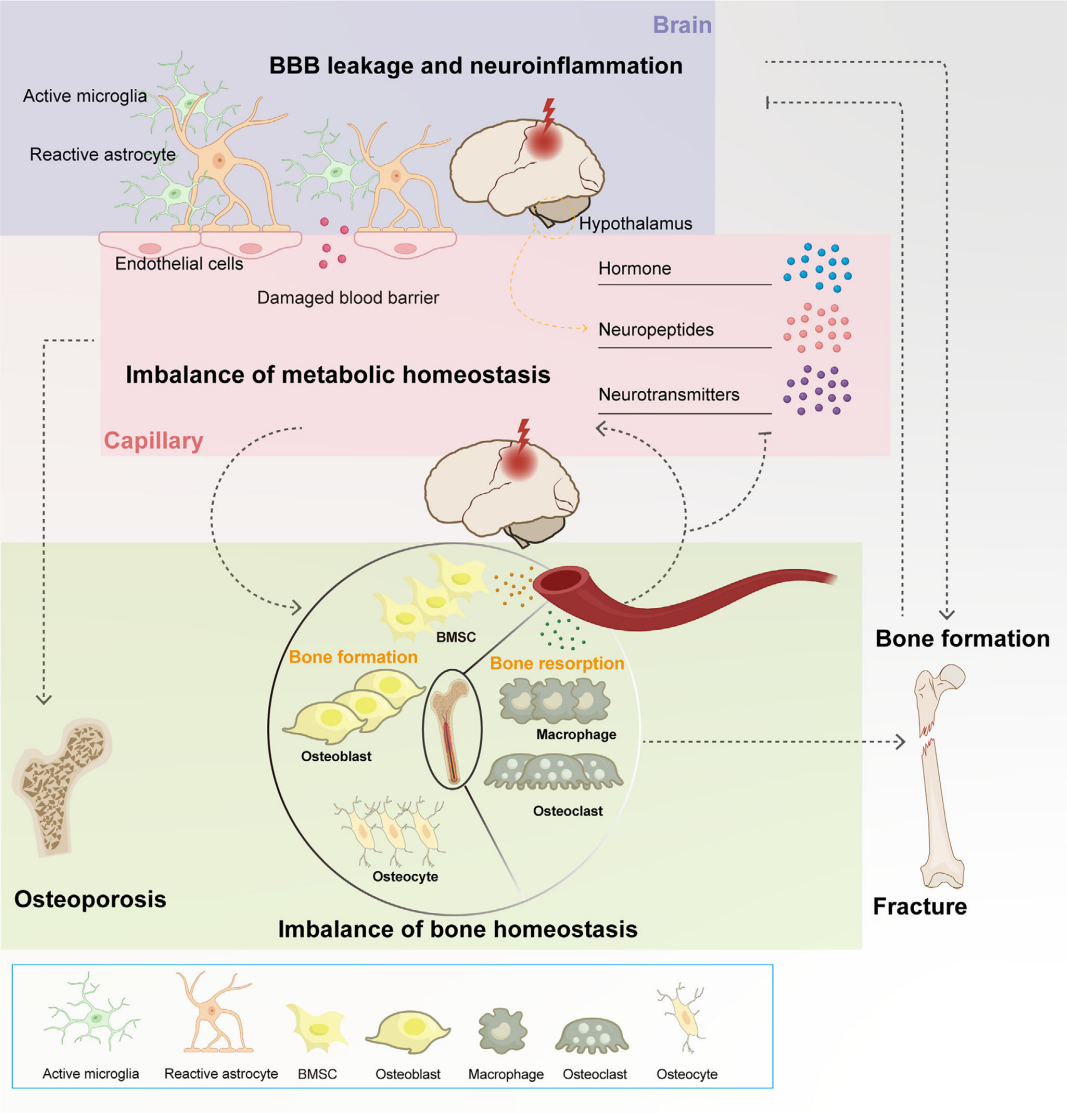
Bidirectional interaction between the brain and bone in traumatic brain injury (TBI). Following TBI, the disruption of the blood–brain barrier and the activation of neuroimmune responses lead to systemic metabolic disturbances, resulting in the abnormal expression of hormones, neuropeptides, and neurotransmitters. One of the significant impacts of TBI is the long‐term loss of bone mass, which may increase the risk of osteoporosis. However, in cases where fractures occur concurrently with TBI, the fracture healing is accelerated, and the osteogenic response is enhanced to facilitate recovery. Conversely, when a fracture occurs, the normal regulatory mechanisms that maintain bone health are disrupted, leading to altered bone formation and bone resorption. This disruption triggers an inflammatory response, whereby pro‐inflammatory cytokines and other mediators released from the injured bone tissue exacerbate the neuroinflammatory processes occurring in the brain following TBI (Solid lines represent microscopic and direct interactions, while dashed lines indicate macroscopic and multi‐pathway relationships.).

TBI not only disrupts normal brain function but also significantly impacts skeletal health. Emerging evidence suggests that TBI can lead to alterations in bone metabolism, resulting in decreased BMD and increased risk of fractures.^[^ ^22^ ^]^ However, the effects of TBI on the skeletal system are complex, as it may contribute to osteoporosis but can also accelerate fracture healing under certain conditions.^[^ ^23^ ^]^ Similarly, the alterations in bone homeostasis and bone metabolism can significantly influence the recovery and outcomes of the brain following TBI. Specifically, bone marrow mesenchymal stromal cells (BMSCs) and bone‐derived factors have been shown to facilitate recovery from TBI,^[^ ^24^ ^]^ highlighting their potential therapeutic role. Existing fractures can exacerbate the outcomes of TBI and create a bidirectional relationship between the brain and bone^[^ ^25^ ^]^ (**Figure** **1**). Understanding these interconnected pathways is crucial, especially considering the rising incidence of TBI and its associated complications. Here we would like to highlight the necessity of communication between CNS and bone, especially the activation of bidirectional brain and bone regulation after TBI. A deeper understanding of the bidirectional communication system between the brain and bone is fundamental to the research of neurological and bone diseases. This review aims to comprehensively explore the multifaceted interactions between TBI and skeletal health and provide novel insights into the study of the brain–bone axis.

## 2 Effects of TBI on Bone

### 2.1 Osteoporosis Occurs After TBI

**Figure 2:**
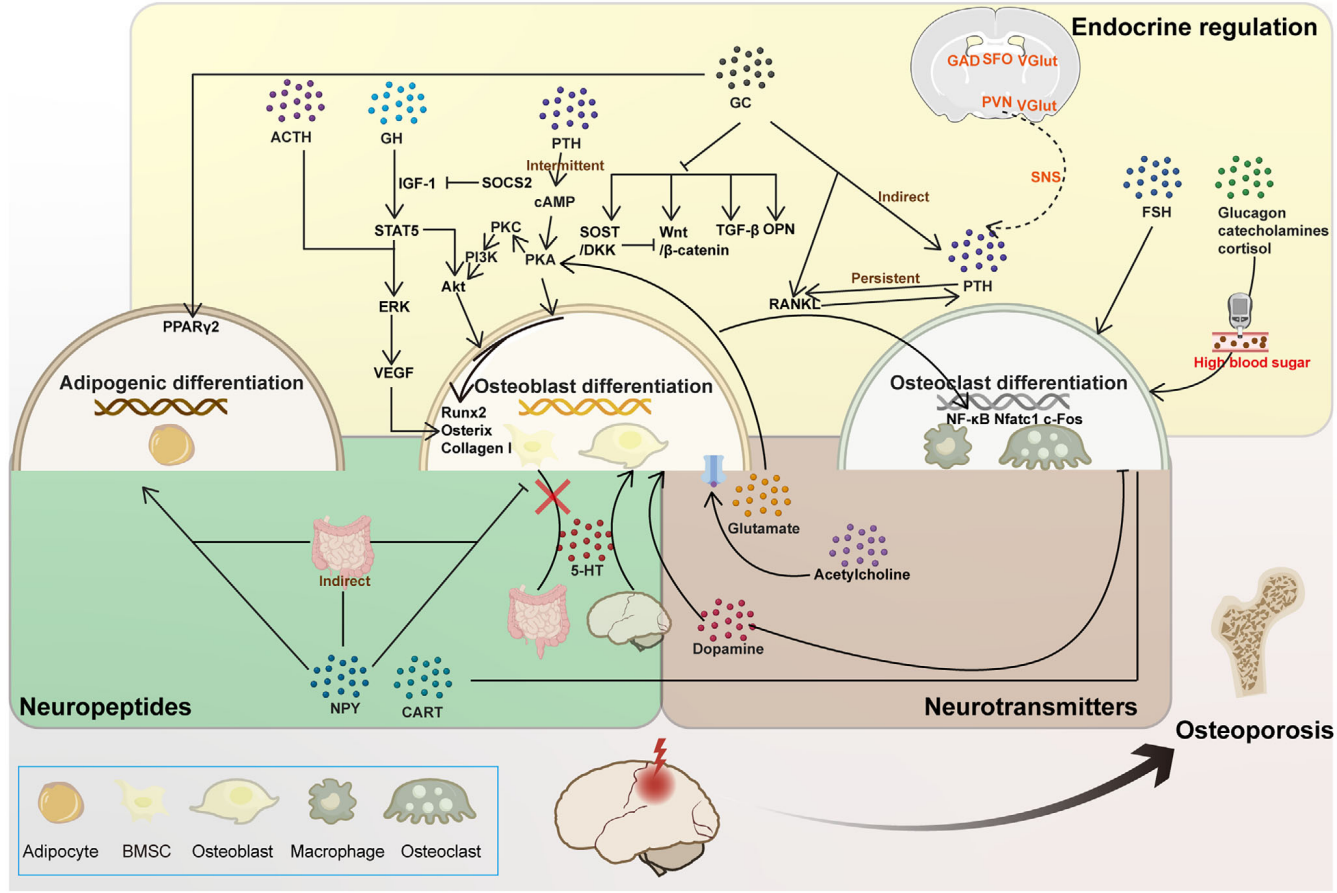
Osteoporosis occurs after TBI. The figure provides an overview of the mechanisms by which TBI contributes to osteoporosis, with a particular focus on the roles of neurohormones, neuropeptides, and neurotransmitters in this process. Following TBI, these bioactive substances regulate bone metabolism, thereby influencing the balance between bone formation and resorption, ultimately leading to a reduction in bone density and an increased risk of fractures (Solid lines represent microscopic and direct interactions, while dashed lines indicate macroscopic and multi‐pathway relationships. Arrows are used to indicate promotion, while lines with horizontal bars represent inhibition.).

The brain is the central control center for tissues and organs and regulates bone metabolism. Damage to the brain from external impact disrupts bone homeostasis through multiple pathways. The Osteoporosis Self‐Assessment Tool for Asians scores decreased in patients with moderate and severe TBI,^[^ ^26^ ^]^ as evidenced by the deterioration of the micro‐architecture of bone tissue and increased bone fragility and high fracture risk.^[^ ^27^, ^28^ ^]^ Further basic medical research confirms these clinical phenomena. Significant bone loss was found in mice with TBI. Micro‐CT results revealed decreased volume and density in cortical bone, decreased number and thickness, and increased spacing in trabecular bone.^[^ ^22^, ^29^ ^]^ The mechanical properties of the femoral and locomotor outcomes in rats with TBI were not different from those of sham groups,^[^ ^30^ ^]^ which suggested that bone loss in TBI was not attributed to immobility. Although immobilization following TBI is a high risk factor for osteoporosis,^[^ ^31^ ^]^ it may be attributed to the fact that mice recover faster after TBI than humans and that humans do not receive appropriate treatment. The above phenomenon is also in line with the current concept of surgery and rehabilitation, which advocates early passive movement and moderate active movement of patients after surgery.^[^ ^32^ ^]^ The negative effects of TBI on bone are well established and include long‐term loss of bone mass and increased risk of osteoporosis (**Figure** **2**).

#### 2.1.1 Neuro‐Endocrine Regulation of TBI on Bone

##### Growth Hormone

Growth hormone (GH) secreted by the anterior pituitary gland is associated with bone metabolism, and GH treatment significantly upregulates the number of osteoblasts in the trabecular bone of mice.^[^ ^33^ ^]^ In this process, GH coupled with Signal transducers and activators of transcription 5 (STAT5) stimulates extracellular regulated protein kinases (ERK) and protein kinase B (Akt) pathways, and the deficiency of insulin‐like growth factor type 1 (IGF‐1) would reduce the activation of STAT5 by GH.^[^ ^34^ ^]^ In addition, suppressor of cytokine signaling 2 (SOCS2) negatively regulates the GH/IGF‐1 pathway, and the knockdown of SOCS2 induces an overgrowth phenotype.^[^ ^35^ ^]^ TBI may lead to permanent or transient pituitary insufficiency with symptoms of GH deficiency,^[^ ^36^, ^37^ ^]^ and GH deficiency tends to be associated with low bone mass.^[^ ^38^ ^]^ Thus, the GH/IGF‐1 axis can be compromised in TBI and, in turn, impact bone growth.

##### Glucocorticoids

Glucocorticoids (GCs) are endogenous steroid hormones produced under extreme stress. The hypothalamic–pituitary–adrenal axis is immediately and profoundly activated in TBI, followed by a massive release of GCs to increase blood pressure and improve the survival rate of patients with TBI.^[^ ^39^ ^]^ Clinically, GCs are often used as a treatment for acute edema and increased intracranial pressure after TBI.^[^ ^40^ ^]^ However, the long‐term treatment of GCs is always associated with significant side effects that can lead to severe disruption of bone homeostasis.^[^ ^41^ ^]^ GCs could enhance bone resorption by promoting osteoclastogenesis through the activation of receptor activator of nuclear factor kappa‐B ligand (RANKL)^[^ ^42^ ^]^ and inhibition of OPN.^[^ ^43^ ^]^ In addition, this effect may be indirectly due to an increase in PTH secretion.^[^ ^42^ ^]^ Overtreatment resulted in lipogenic differentiation in BMSCs accompanied by up‐regulation of peroxisome proliferator‐activated receptor γ2 (PPARγ2), adipsin, and CCAAT enhancer‐binding proteins (C/EBPs).^[^ ^44^ ^]^ Meanwhile, osteogenesis was inhibited by GCs, resulting in decreased bone formation through transforming growth factor‐β (TGF‐β)^[^ ^45^ ^]^ and Wnt/β‐catenin signaling pathways.^[^ ^46^, ^47^ ^]^ Wnt inhibitors are upregulated during glucocorticoid overexpression, thereby impeding osteogenesis.^[^ ^46^ ^]^ GCs were irregularly secreted due to HPA axis disorders or the therapeutic use of prolonged GCs after TBI and, in turn, affected osteogenesis and bone resorption, further deteriorating the metabolic status of the bone, and ultimately leading to osteoporosis^[^ ^41^ ^]^

##### Adrenocorticotrophic Hormone

A significant proportion of patients present with sustained adrenocorticotropic hormone (ACTH) deficiency after TBI due to the damage to the pituitary gland, and this condition can lead to persistent cognitive or behavioral disorders and impairments in activities of daily living.^[^ ^48^ ^]^ Drugs used for treatment in the acute phase also induce changes in cortisol metabolism.^[^ ^49^ ^]^ Although ACTH deficiency may subside as the damaged pituitary recovers after TBI, patients after 5 months of recovery may again experience symptoms of ACTH deficiency.^[^ ^50^ ^]^

High‐affinity melanocortin receptors detected in osteoblasts could bind to ACTH to promote osteogenesis.^[^ ^51^ ^]^ Osteoblast‐specific markers, such as runx2, osterix, and collagen I, were upregulated with ACTH treatment, suggesting the importance of ACTH in promoting osteogenic differentiation and maintaining bone mass. ACTH activates ERK signaling and promotes the production of vascular endothelial growth factor‐A (VEGF) in developing osteoblasts, which augments bone formation and alkaline phosphatase activity.^[^ ^52^ ^]^ Disorders of ACTH are related to bone homeostasis. However, biochemical diagnosis of adrenal insufficiency is difficult in cases of critical illnesses such as TBI, because the critical value of this indicator has not been well defined.^[^ ^49^ ^]^ Nevertheless, the effects of fluctuating changes in ACTH on bone after TBI are well established. Therefore, an imbalance in ACTH metabolism is likely to be involved in the development of osteoporosis in patients with TBI.

##### Parathyroid Hormone

PTH is one of the most important hormones for bone turnover and calcium homeostasis because it is involved in the regulation of bone mineral homeostasis and biological developmental processes.^[^ ^53^ ^]^

In persistent hyperparathyroidism‐caused bone loss, osteocytes are the direct target of PTH, and the action of PTH promotes RANKL expression in osteocytes to increase osteoclast number and function.^[^ ^54^ ^]^ Interestingly, intermittent PTH increases bone mass because of the ability of PTH to activate cAMP‐mediated PKA and anti‐apoptotic signaling pathways, accompanied by an increase inf Runx2, which enhances osteoblast survival and osteogenic differentiation.^[^ ^55^ ^]^ Thyrotropin deficits were particularly common in patients with TBI.^[^ ^48^ ^]^ The hypothalamic–pituitary–thyroid axis may disrupt the regulation of systemic bone homeostasis in TBI. Chemogenetic stimulation of GABAergic projections from the SFO to the PVN decreased serum PTH and trabecular bone mass and stimulated glutamatergic neurons to promote serum PTH and bone mass.^[^ ^14^ ^]^ These findings provide novel insights into central nervous regulation of PTH at the cellular and circuit levels.

##### Glucagon and Insulin

Hyperglycemia manifests in numerous patients with TBI in clinical practice and is considered a predictor of poor neurological prognosis.^[^ ^56^ ^]^ Diabetic individuals with hyperglycemia and insulin resistance had a significantly higher risk of death after TBI.^[^ ^57^ ^]^ The activation of the hypothalamic–pituitary–adrenal axis and the sympathetic autonomic nervous system by TBI led to an upregulation of the levels of glucagon, catecholamines, and cortisol, which greatly increased blood glucose levels.^[^ ^58^ ^]^ Diabetes decreased bone mass and strength and increased bone fragility through cellular abnormalities, matrix interactions, immune and vascular changes, and musculoskeletal maladaptation to chronic hyperglycemia.^[^ ^59^ ^]^ Nevertheless, the potential relationship between dysglycemia after TBI and osteoporosis lacks further study and may provide a new therapeutic direction.

##### Gonadotropins

The gonadotropin axis is suppressed in critical illness, and the severity of TBI is strongly associated with hypogonadism.^[^ ^60^ ^]^ High expression of follicle‐stimulating hormone (FSH) led to hypogonadal bone loss by enhancing osteoclastic differentiation and bone resorption.^[^ ^61^ ^]^ However, similar disturbances in FSH and testosterone have been observed in non‐head injury patients, suggesting that these hormones may not be specific to head injury but are more likely to be associated with general stress in critical illness.^[^ ^37^ ^]^ Children and adolescents taking gonadotropin‐releasing hormone may be at risk for decreased BMD.^[^ ^62^ ^]^ Research is needed to determine the clinical significance of changes in bone density in relation to fracture risk.

#### 2.1.2 Neuropeptides of TBI on Bone

##### Neuropeptide Y

Neuropeptide Y (NPY), an endogenous active substance, is involved in a variety of pathophysiological processes. In CNS, NPY is distributed in the amygdala, locus coeruleus, and cerebral cortex, with the highest expression levels in the hypothalamus.^[^ ^63^ ^]^ The expression of NPY was not different in animals with mild brain injury, but significant changes in NPY expression were observed in the hippocampus of animals with severe brain injury.^[^ ^64^ ^]^ Furthermore, only slight changes in NPY fluctuated in the early stages of brain injury, whereas in the later stages, the increase in NPY was significant.^[^ ^65^ ^]^ The expression of NPY and its receptor was monitored in osteoblasts, osteocytes, and adipocytes.^[^ ^66^ ^]^ Previous studies confirmed that NPY can strongly influence bone metabolism in a direct and indirect manner. Despite the paradoxical role of NPY in the maintenance of skeletal homeostasis,^[^ ^67^ ^]^ it plays a predominantly negative role in the progression of osteoporosis. NPY inhibited osteogenesis and promoted adipogenesis in BMSCs, and its expression increased with age and led to the progression of osteoporosis.^[^ ^66^ ^]^ Additionally, exogenous overexpression of NPY exacerbated postmenopausal osteoporosis by modulating intestinal flora,^[^ ^68^ ^]^ and specific overexpression of NPY in the hypothalamus induced an obese phenotype and a significant reduction in bone mass.^[^ ^69^ ^]^ NPY in the hypothalamus regulates skeletal homeostasis and fat metabolism via neuroendocrine descending interoception.^[^ ^70^ ^]^ Overall, NPY is likely to be involved in the pathogenesis of osteoporosis after TBI.

##### Cocaine Amphetamine Regulates Transcript

Sustained decrease occurred in the levels of acetylated histone H3‐Lys 9 in promoter region of the cocaine amphetamine regulated transcript (CART) gene in repeated mild TBI concomitant with a decrease in the levels of CART mRNA and peptide.^[^ ^71^ ^]^ The knockout or reduction of CART in mice exhibited bone loss by increasing the number of osteoclasts to promote bone resorption.^[^ ^72^, ^73^ ^]^ However, an increase in peripheral circulation rather than injection of exogenous CART into the third ventricle rescued the low bone mass in CART^−/−^ mice.^[^ ^72^ ^]^

#### 2.1.3 Neurotransmitters on Bone in TBI

##### Neurotransmitters Released by CNS

Neurotransmitters released by the CNS are associated with bone homeostasis and bone metabolism. Localized injuries cause cellular release of excess glutamate, an excitatory neurotransmitter that is neurotoxic.^[^ ^74^ ^]^ Numerous studies have shown the dysregulation of glutamate in patients and animals with TBI.^[^ ^75^ ^]^ N‐methyl‐D‐aspartate (NMDA) receptor was detected in osteoblasts^[^ ^76^ ^]^ and stimulated osteoblasts differentiation through protein kinase A (PKA), protein kinase C (PKC), and phosphoinositide 3‐kinase (PI3K) signaling pathways.^[^ ^77^ ^]^ Nevertheless, the modulation between osteoporosis in TBI and dysregulation of glutamate still lacks clear evidence.

5‐Hydroxytryptamine (5‐HT) is a well‐known hormone and neurotransmitter that is primarily produced by enterochromaffin cells in the gut (95%), as well as in neurons of the brain stem (5%).^[^ ^78^, ^79^ ^]^ The involvement of the 5‐HT system in bone metabolism remains controversial. 5‐HT treatment increased bone formation in mice^[^ ^80^, ^81^ ^]^ while gut‐derived serotonin had no effect on BMD.^[^ ^82^ ^]^ Those studies suggest that the role of 5‐HT in bone homeostasis may be analyzed specifically according to specific physiological or disease states, especially TBI.

The neurotransmitter dopamine is also implicated in bone homeostasis. TBI inhibited dopamine release and reuptake in the acute and subacute phases after TBI.^[^ ^83^ ^]^ Dopamine and dopamine D2‐like receptor agonists inhibited osteoclast differentiation in cells of human and mouse origin.^[^ ^84^ ^]^ In addition, the expression of dopamine receptors in BMSCs was upregulated during osteogenic differentiation, and osteogenic mineralization was significantly increased following dopamine treatment.^[^ ^85^, ^86^ ^]^ Targeting dopaminergic signaling is important for adjusting bone homeostasis after TBI.

##### Neurotransmitters Released by the Autonomic Nervous System

Acetylcholine serves as the main neurotransmitter of parasympathetic nerves. Studies have shown that inhibition of cholinergic neurotransmission decreased the release of acetylcholine.^[^ ^87^ ^]^ Degenerative changes in autonomic nerves were associated with the development of osteoporosis.^[^ ^88^ ^]^ Muscarinic and nicotinic acetylcholine receptors (mAChR and nAChR) were expressed in osteoblasts.^[^ ^89^ ^]^ mAChR M3R stimulated cancellous bone microarchitecture, bending stiffness, and bone matrix synthesis, and was a positive regulator of increased bone mass.^[^ ^90^, ^91^ ^]^ Flexural stiffness and maximum breaking force were significantly reduced in nAChR subunit α9 KO mice. The rate of osteocyte degeneration and death increased without significant effects on osteoclasts.^[^ ^92^ ^]^ However, osteoclastogenesis was reduced in α7 KO mice.^[^ ^17^ ^]^

Norepinephrine is the main neurotransmitter of sympathetic nerves^[^ ^93^ ^]^ and is involved in the regulation of bone homeostasis.^[^ ^94^ ^]^ MicroRNA‐21 was upregulated in osteoblasts due to the activation of β adrenergic receptors (βARs) and stimulates osteoclasts via exosomal transport to induce osteoporosis.^[^ ^95^, ^96^ ^]^ Hence, the dysregulation of sympathetic activity in TBI may contribute to bone loss.

#### 2.1.4 Other Factors

The skeletal system, regulated by the nervous system, responds to external stimuli and undergoes physiological and pathological changes. Neuro‐skeletal system interactions affect bone morphology, density, and quality. Muscle atrophy, reduced physical activity, and lack of nutrition are common sequelae in patients with TBI.^[^ ^97^ ^]^ These factors can overuse the skeletal system, which in turn leads to the development of osteoporosis. In addition, mechanical factors caused by positional changes, reduced weight support, and poor mattress selection can lead to accelerated bone loss, osteoporosis, and a host of other problems.^[^ ^28^, ^98^ ^]^ Therefore, timely monitoring and comprehensive treatment are necessary in patients with TBI.

### 2.2 TBI Accelerates Bone Healing

Fractures are the most common organ injuries. The fracture healing process is influenced by the patient's systemic condition (e.g., age, nutritional and hormonal metabolic balance), changes in the local microenvironment, and mechanical stress. Most fractures that are treated return to normal structure and function in the current medical environment. However, delayed union and nonunion still occur in 5% to 10% of patients worldwide and increase with the severity of trauma, age, and basal metabolic status.^[^ ^99^ ^]^ Fracture non‐union greatly increases the economic, psychological, and life burdens of patients and reduces their quality of life.^[^ ^100^ ^]^ Therefore, research to promote bone regenerative repair and accelerate fracture healing is crucial.

**Figure 3:**
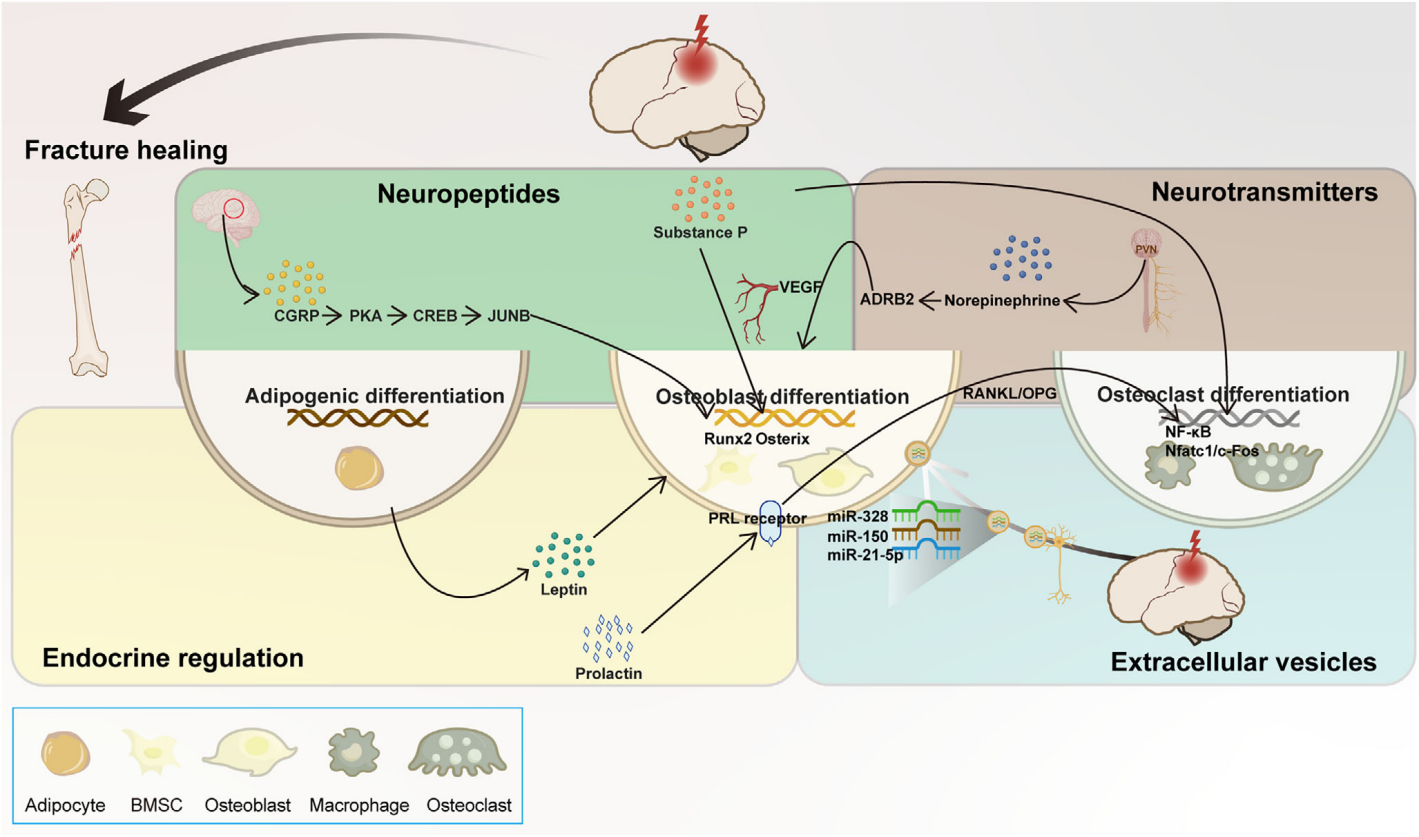
TBI accelerates bone healing. The figure outlines the impact of TBI on fracture healing, with neurohormones, neuropeptides, neurotransmitters, and extracellular vesicles. It illustrates how the interactions between the nervous and endocrine systems following TBI influence bone metabolism, including alterations in the balance between bone formation and resorption, thereby promoting the formation of regenerated bone tissue.

Interestingly, TBI can accelerate fracture healing, and increasing lines of evidence confirm an association between TBI and enhanced post‐fracture osteogenesis. Clinically, it was first reported by Gibson and Roberts successively in the 1960s that they found a significant acceleration of fracture healing and a significant increase in the amount of bone scab formation in patients with TBI,^[^ ^101^, ^102^ ^]^ since then, numerous studies have investigated the relationship between TBI and fracture healing, and the acceleration of fracture healing after TBI has gradually been recognized. Central nerve injury promoted bone scab formation and accelerated fracture healing; thus, the regulatory role of neurological factors on fracture healing is certain. Notably, clinical studies have reported a significantly faster rate of fracture healing and increased scab formation in patients with combined TBI compared to patients with a simple fracture.^[^ ^103^, ^104^ ^]^ Multiple neuropeptides, hormones, and exosomes were released in TBI by regulating the systemic metabolic system,^[^ ^105^ ^]^ which in turn accelerated bone healing. Here, we summarize existing studies (**Figure** **3**) and hope to provide new research directions and clinical treatments for the treatment of osteogenesis imperfecta, as well as cognitive recovery after TBI.

#### 2.2.1 Endocrine Regulation

Leptin is secreted by adipocytes and is closely related to the regulation of body feeding, energy metabolism, fat storage, and bone homeostasis.^[^ ^106^ ^]^ Leptin enhances hypothalamic glucose sensing and restores glucose homeostasis in uncontrolled diabetic conditions.^[^ ^107^ ^]^ Leptin plays an important role in fracture healing and bone formation. Leptin deficiency retarded the rate of fracture healing in mice after TBI.^[^ ^108^, ^109^ ^]^ The findings suggest that the increase in healing tissue formation after TBI may be dependent on leptin signaling.

Prolactin (PRL) receptors were identified in osteoblasts, suggesting a possible direct action of PRL on bone.^[^ ^110^ ^]^ Sustained pathological PRL exposure stimulated bone turnover and induced osteoporosis.^[^ ^111^, ^112^ ^]^ Prolactin regulates bone homeostasis by increasing RANKL and decreasing OPG expression in osteoblasts, thereby inhibiting osteoclast proliferation and bone mineralization.^[^ ^110^ ^]^ Patients with consolidated fractures and TBI had significantly higher blood levels of PRL at week 5 post‐trauma.^[^ ^113^ ^]^ Thus, PRL not only influences the physiology of bone metabolism but also appears to be one of the humoral factors involved in osteogenic enhancement in patients with TBI.

#### 2.2.2 Neuropeptides

CGRP is a short half‐life pro‐osteogenic neurotransmitter. Higher expression of CGRP was found in the hippocampus following TBI, and CGRP accelerated fracture healing in TBI.^[^ ^114^ ^]^ Specifically, CGRP enhanced osteogenic differentiation of BMSCs through the PKA/cAMP response element‐binding protein (CREB)/JUNB pathway to induce the activation of osterix.^[^ ^115^, ^116^, ^117^ ^]^ This provides new insights into revealing TBI‐accelerated fracture healing as well as different perspectives on the brain–bone axis after TBI.

Substance P is a neuropeptide belonging to the tachykinin family and is involved in neuroinflammation, increased blood‐brain barrier permeability, and edema formation in TBI.^[^ ^118^ ^]^ Persistent high levels of substance P in the serum of patients with severe head injury at an early stage were associated with increased mortality.^[^ ^118^ ^]^ Substance P binds to neurokinin‐1 receptors after TBI, leading to neurogenic inflammation that increases intracranial pressure and has neurotoxic effects.^[^ ^119^ ^]^ The area covered by hypertrophic chondrocytes was reduced in SP‐deficient mice (Tac1^−/−^), suggesting that SP deficiency delayed the terminal differentiation of hypertrophic chondrocytes.^[^ ^120^, ^121^ ^]^ SP is essential for the process of chondral osteogenesis in the fracture healing process.

#### 2.2.3 Autonomic Nervous System

TBI caused dysregulation of the systemic immune response,^[^ ^122^ ^]^ and accelerated bone healing by activating the PVN of the hypothalamus, which elevated sympathetic tone to promote myelopoiesis and M2 polarization in macrophages at the fracture site.^[^ ^104^, ^123^ ^]^

Norepinephrine is a key neurotransmitter with important roles in the control of the cardiovascular system and metabolism. In the acute phase after TBI, pressure stimulation led to activation of the sympathetic nervous system and a massive release of norepinephrine. Fracture nonunion occurred in adult mice lacking β2‐adrenergic receptor (ADRB2) without TBI. Norepinephrine stimulated the expression of VEGF and CGRP in periosteal cells via ADRB2 and thereby promoted osteogenic H‐vessel formation in the fracture scab after TBI.^[^ ^124^ ^]^

#### 2.2.4 Extracellular Vesicles

Extracellular vesicles (EVs) encompass various subtypes of membranous structures released by cells, including exosomes, microvesicles, microparticles, apoptotic vesicles, and others.^[^ ^125^ ^]^ The lipid bilayer separates the contents of EVs from the fluid‐based extracellular environment and helps to maintain vesicle stability.^[^ ^126^ ^]^ EVs can circulate through body fluids such as blood, reaching target cells in distant organs, thereby facilitating communication and interaction between organs. The microRNAs contained within EVs can regulate cellular functions by inhibiting the expression of target genes. These miRNAs diffuse to peripheral tissues via EVs, influencing the biological functions of remote organs or tissues. EVs derived from adult AD mice brains significantly impact the differentiation of BMSCs, leading to an imbalance in bone metabolism. Specifically, miR‐483‐5p exerts its effects by inhibiting the expression of Igf2, thereby suppressing osteogenic differentiation and promoting adipogenic differentiation, which contributes to the development of osteoporotic in AD mice.^[^ ^127^ ^]^ Conversely, EVs derived from osteoblasts in young mice can be transported to the brain, reducing amyloid‐beta deposition and neuronal apoptosis, consequently improving cognitive function. However, this protective effect diminishes with age.^[^ ^128^ ^]^ Furthermore, microRNA‐146a present in BMSC‐derived extracellular vesicles can act on astrocytes to exert anti‐inflammatory effects.^[^ ^129^ ^]^ This highlights the critical role of extracellular vesicles in inter‐organ communication, including the bone–brain axis.

Brain‐derived EVs are important carriers for the distant exchange of cells between the brain and other organs. EVs were involved as messengers in brain–lung crosstalk^[^ ^130^ ^]^ and neurogenic heterotopic ossification^[^ ^131^ ^]^ after TBI. Notably, brain‐derived EVs play an active role in fracture healing. The injured neurons in TBI released osteogenic miRNA‐enriching small EVs, which were transferred to bone through the circulatory system, and targeted osteoprogenitors to promote osteogenesis and accelerate bone healing.^[^ ^132^ ^]^ Plasma exosomes after TBI had the ability to promote osteoblast proliferation and differentiation, and sequencing revealed that a large number of osteoblast‐related miRNAs are increased in exosomes.^[^ ^133^ ^]^ It has also been found that miR‐21‐5p is significantly enriched in TBI‐Exosomes, which could be delivered to human mesenchymal stem cells and combined with SMAD7 to enhance osteoblast differentiation and fracture healing.^[^ ^134^ ^]^ Together, these studies establish a clear link between damaged brain and bone formation in animal and clinical settings, providing an avenue for bone‐targeted therapy.

#### 2.2.5 Other Factors

Large amounts of cytokines and growth factors are released in patients with TBI and can play an important role in accelerating fracture healing. Serum levels of bone morphogenetic protein 2 (BMP‐2), fibroblast growth factor 2 (FGF‐2), interleukin 1 beta (IL‐1β) and platelet‐derived growth factor (PDGF) were found to be significantly elevated in patients with femur fractures accompanied by TBI injury, which may be involved in the process of bone healing being accelerated.^[^ ^135^ ^]^ In addition, accelerated fracture repair in patients with TBI may be associated with increased serum concentrations of hypoxia‐inducible factor‐1α.^[^ ^136^ ^]^ The number of mast cells and the expression of CXCL10 were significantly decreased in fracture mice combined with TBI. These led to down‐regulation of osteoclastogenesis and inhibition of bone resorption and remodeling.^[^ ^122^ ^]^ Mesenchymal stem cells recruited by stromal‐derived factor 1 participated in endochondral bone repair under the condition of TBI.^[^ ^137^ ^]^ Cannabinol‐1 receptor‐mediated osteoanabolic responses were found to be involved in skull defect healing after mild TBI.^[^ ^138^ ^]^

## 3 Bone to Brain in TBI

The interaction between the skeletal system and the brain is complex, and the influence of the skeleton on the brain becomes particularly evident following TBI. BMSCs possess the capacity for self‐renewal and multipotent differentiation. In the early intervention phase after brain injury, BMSCs promote neural repair and inhibit inflammatory responses by releasing bioactive factors, such as cytokines and exosomes, thereby improving neurological function and facilitating neuronal regeneration. Additionally, bone tissue secretes various bioactive factors, including bone morphogenetic proteins (BMPs) and osteopontin (OPN), which play crucial roles in neural repair. These factors can enhance the survival and regeneration of neurons and modulate the polarization states of microglia, thereby alleviating inflammatory responses and actively promoting the recovery from brain injuries.

**Figure 4:**
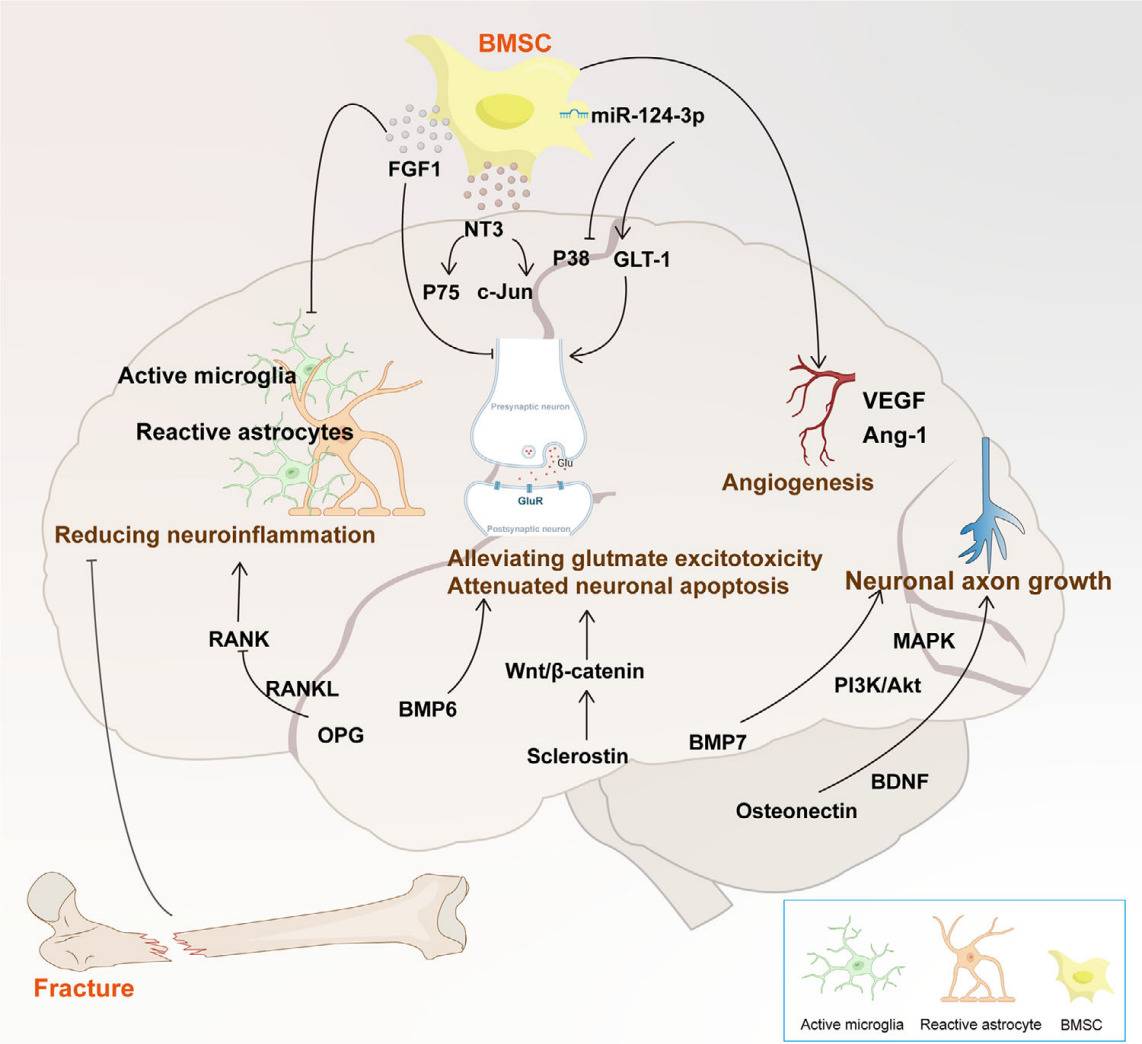
Bone to brain in TBI. Bone marrow mesenchymal stem cells (BMSCs) and bone tissue play a crucial role in promoting neural repair through the release of cytokines, exosomes, and other bioactive factors. These factors contribute not only to the enhancement of neurological function and the regeneration of neurons but also to the modulation of microglial cell polarization, thereby alleviating inflammatory responses and actively facilitating recovery following TBI. However, the imbalance of bone homeostasis following a fracture may exacerbate the inflammation and edema associated with TBI, creating a detrimental cycle that can negatively impact neural repair. Therefore, maintaining bone homeostasis is of paramount importance in the recovery process following TBI. (Arrows are used to indicate promotion, while lines with horizontal bars represent inhibition.).

Research has shown that patients with fractures may experience more severe neurological damage when undergoing brain injury, potentially due to systemic inflammatory responses triggered by the fracture. The release of inflammatory factors may exacerbate damage to brain tissue. Furthermore, fractures may affect patients' mobility and recovery processes, further influencing the recovery from brain injury. In summary, the bone–brain axis plays a significant role in the recovery from TBI (**Figure** **4**), indicating that clinical treatment should comprehensively consider the relationship between the skeletal and nervous systems.

### 3.1 Effects of BMSCs in TBI

BMSCs have been extensively investigated for their application in neurological disorders. Due to the multipotent differentiation capacity, immunomodulatory properties, and neuroprotective effects, BMSCs have been utilized as a promising regenerative therapeutic strategy and have emerged as a novel target in the research of TBI treatment.^[^ ^139^ ^]^

#### 3.1.1 Direct Effects

In the early stages of acute brain injury, injured cells release a series of harmful signals that trigger an immune response. Microglia and astrocytes rapidly respond to this immune reaction. The activation of microglia and the polarization of astrocyte were inhibited by BMSCs.^[^ ^24^, ^140^ ^]^ The application of ectoderm‐derived frontal bone mesenchymal stem cells significantly alleviated neuroinflammation and excitotoxicity to neurons in mice with TBI, resulting in improvements in cognitive and behavioral deficits.^[^ ^140^ ^]^ Significantly, phase 2 clinical trials have demonstrated that an intravenously delivered autologous bone marrow mononuclear cell infusion for the treatment of severe TBI in adults was safe and feasible, and effectively reduced key inflammatory biomarkers.^[^ ^141^ ^]^ This suggests that BMSC transplantation for the treatment of TBI is promising and predictable.

Angiogenesis can improve tissue ischemia by providing oxygen and nutrients, thereby promoting structural remodeling of damaged brain tissue and accelerating the repair of neural function. The upregulation of the expression of VEGF and Ang‐1 due to the transplantation of BMSCs promoted the formation of micro‐vessels and improved neurological function.^[^ ^142^ ^]^ This finding provides new insights into BMSC‐based treatment of TBI.

#### 3.1.2 Indirect Effects

BMSCs exert a neuroprotective effect on TBI by secreting neurotrophic factors. BMSCs migrated to the injured area after TBI and differentiated into neurons and astrocytes. Multiple neurotrophic factors were increased to enhance motor function recovery after TBI.^[^ ^143^ ^]^ Neurotrophic factor 3 (NT3) was one of the key factors in BMSC‐mediated neurological recovery. NT3^P75‐2^ significantly improved neurological recovery after TBI by increasing the survival rate of BMSCs and ameliorating the inflammatory environment through the P75 neurotrophic factor receptor and the c‐Jun N‐terminal kinase signaling pathway.^[^ ^24^ ^]^ In addition, FGF1 secreted by frontal BMSCs enhanced glutamate transport by astrocytes and attenuated the cytotoxic effects of excess glutamate on neurons.^[^ ^140^ ^]^ These molecules enhance the survival and regeneration of nerve cells while effectively reducing apoptosis and tissue damage. Additionally, they improve the microenvironment of brain tissue, facilitating nerve repair and mitigating secondary pathological changes following injury.

Exosomes derived from BMSCs show great promise in the treatment of brain diseases. The polarization of microglia/macrophages is inhibited by the early administration of BMSC‐derived exosomes following TBI.^[^ ^144^ ^]^ Exosomes enriched with miR‐124‐3p attenuated glutamate‐mediated excitotoxicity by down‐regulating p38 / mitogen‐activated protein kinase (MAPK) and up‐regulating GLT‐1 expression, thereby reducing neurological damage in TBI.^[^ ^145^, ^146^ ^]^ EVs derived from hypoxic mesenchymal stem cells can promote angiogenesis, brain remodeling, and neural recovery following focal cerebral ischemia in mice.^[^ ^147^ ^]^ BMSC‐derived EVs exert a neuroprotective effect against neuronal damage induced by intracerebral hemorrhage through the regulation of ferroptosis via miR‐214‐3p.^[^ ^148^ ^]^ Additionally, MSC‐EVs enhance angiogenesis through the upregulation of VEGF and CXCR4, resulting in a reduction of infarct volume and an improvement in motor function in mice.^[^ ^149^ ^]^ EVs derived from BMSCs have been shown to enhance cognitive function in mice while alleviating microglial activation and the occurrence of inflammation.^[^ ^150^ ^]^ Administration of BMSC‐derived EVs into the lateral ventricle suppresses the hyperactivation of microglia and astrocytes in the hippocampus, accompanied by a reduction in pro‐inflammatory cytokines such as IL‐1β, IL‐6, and TNF‐α, as well as an upregulation of synaptic‐related proteins and brain‐derived neurotrophic factors (BDNF).^[^ ^151^ ^]^ Moreover, BMSC‐derived EVs deliver microRNA‐29c‐3p to neurons, which inhibits the expression of beta‐secretase 1 and activates the Wnt/β‐catenin signaling pathway, thereby improving cognitive function in AD mice.^[^ ^152^ ^]^ Due to the excellent biocompatibility and low immunogenicity, BMSC‐derived exosomes show significant potential for clinical applications. Future studies can further investigate their application in TBI and other neurological diseases to provide new treatment options for patients.

### 3.2 Effects of Bone‐Derived Factors in TBI

#### 3.2.1 Bone Morphogenetic Proteins

BMPs belong to the transforming growth factor‐beta (TGF‐β) superfamily and are crucial for the lineage specification and differentiation of osteoblasts and chondrocytes, as well as for skeletal development and homeostasis.^[^ ^153^ ^]^ BMPs play a significant role in the regulation of systemic bone metabolism. Furthermore, BMP signaling is essential for the development and maintenance of both the CNS and the PNS. The BMP family, along with its numerous receptors, cofactors, and downstream signaling pathways, negatively regulates neurogenesis, actively promotes gliogenesis, influences the stem cell reservoir, and modulates synaptogenesis and axon growth.^[^ ^154^ ^]^ Pre‐treatment with BMP6 has been shown to enhance motor function in rats and to mitigate apoptosis, thereby reducing reperfusion‐induced cerebral infarction.^[^ ^155^ ^]^ Additionally, the expression of BMP7 is upregulated following cerebral ischemic injury. Treatment with BMP7 has been found to correct body asymmetry and neurological deficits, as well as to promote neuronal regeneration after stroke.^[^ ^156^ ^]^ Through promoting neuronal regeneration and modulating inflammatory responses, BMPs offer novel therapeutic avenues for improving recovery in patients with neurological disorders. Future research could further explore the potential applications of BMPs in TBI treatment, providing additional evidence to support clinical interventions.

#### 3.2.2 Osteopontin

OPN is a multifunctional extracellular matrix protein that plays a critical role in bone metabolism and homeostasis. It is closely associated with the onset and progression of various skeletal disorders, including osteoarthritis and osteoporosis.^[^ ^157^ ^]^ Elevated levels of OPN have been linked to low BMD and osteoporotic vertebral fractures in postmenopausal women.^[^ ^158^ ^]^ Furthermore, the expression of OPN in plasma may serve as a predictive biomarker for the severity of neuroinflammation and TBI in children.^[^ ^159^ ^]^ During the early stages of ischemia, the expression of OPN is notably upregulated, particularly in microglia, and is associated with lysosomal damage, inflammasome activation, and autophagosome accumulation. Knockdown of the expression of OPN has been shown to mitigate ischemic brain injury in mice.^[^ ^160^ ^]^ OPN is activated in CD11c‐positive microglia in AD, and inhibiting its production can reduce the number of pro‐inflammatory microglia, plaque formation, and atrophic neurites, thereby improving cognitive function.^[^ ^161^ ^]^ Given its multifaceted roles, OPN is considered a potential therapeutic target, and future research may further investigate its applications in the treatment of bone and neural injuries.

#### 3.2.3 Osteocalcin

Osteocalcin (OCN) is a non‐collagenous protein synthesized by osteoblasts that plays a crucial role in bone mineralization and metabolism.^[^ ^162^ ^]^ OCN is not only linked to bone health but may also exert effects on the CNS and conditions such as TBI. Osteoblasts utilize osteocalcin to enhance glucose metabolism, insulin sensitivity, and energy expenditure, thereby maintaining metabolic homeostasis. OCN can cross the blood–brain barrier (BBB), primarily existing in its uncarboxylated form.^[^ ^163^ ^]^ Following TBI, OCN is highly specifically expressed in neutrophils within the cranial bone marrow, where it reprograms glucose metabolism through the downregulation of the glycolytic‐related enzyme glyceraldehyde‐3‐phosphate dehydrogenase, which provides neuroprotective effects.^[^ ^164^ ^]^ The synthesis and release of OCN are diminished in leptin‐deficient mice, which impairs the accelerated fracture healing process observed in TBI.^[^ ^165^ ^]^ Interestingly, the absence of OCN leads to significant deficits in spatial learning and memory and exacerbates anxiety‐like behavior.^[^ ^166^ ^]^ Additionally, osteoblast‐specific OCN knockout mice exhibit reduced testis size and body weight, alongside diminished spermatocyte numbers and increased apoptosis of germ cells.^[^ ^167^ ^]^ These findings underscore the importance of OCN in maintaining metabolic homeostasis, normal reproductive function, and motor abilities. However, this field remains in its exploratory stages, and future research is needed to further elucidate the specific mechanisms of OCN action and its potential clinical applications.

#### 3.2.4 Sclerostin

Sclerostin, primarily produced by osteocytes, acts as a paracrine regulator of osteoblast and osteoclast activity on the bone surface through Wnt signaling pathways.^[^ ^162^ ^]^ Elevated levels of sclerostin have been detected in the serum following acute ischemic stroke.^[^ ^168^ ^]^ Furthermore, increased serum levels of sclerostin may serve as a biomarker for identifying elderly individuals at high risk for cognitive impairment, aiding in the detection of preclinical AD.^[^ ^169^ ^]^ Sclerostin, derived from osteocytes can cross the blood–brain barrier in aged mice, and abnormally high levels of sclerostin impair synaptic plasticity and memory in these mice by inhibiting the Wnt/β‐catenin signaling pathway.^[^ ^170^ ^]^ Additionally, the intracerebroventricular injection of sclerostin induces anxiety‐like behaviors, reduces social hierarchy, and decreases the dendritic complexity of hippocampal pyramidal neurons in mice.^[^ ^171^ ^]^ These findings suggest that osteocyte‐derived sclerostin‐mediated bone–brain crosstalk could serve as a potential target for therapeutic interventions in neurodegenerative diseases.

#### 3.2.5 Osteonectin

Osteonectin is abundant in bone and is highly expressed in areas of active remodeling outside of the bone. Mice lacking osteonectin show a downregulation of osteoblasts and osteoclasts, leading to reduced bone formation and decreased bone remodeling, which in turn results in an imbalance in bone homeostasis and metabolic disorders.^[^ ^172^ ^]^ Additionally, osteonectin is secreted by Schwann cells, where it plays a role in regulating neuronal axon growth.^[^ ^173^ ^]^ It works synergistically with BDNF to promote the growth of retinal ganglion cells through the PI3K/Akt and MAPK signaling pathways.^[^ ^174^ ^]^ These findings suggest that osteonectin may have potential roles in neuroinflammation and neurodegenerative diseases.

#### 3.2.6 Osteoprotegerin

Osteoprotegerin (OPG) is a key regulatory factor in bone metabolism, primarily functioning to reduce bone resorption by inhibiting the formation and activity of osteoclasts. Decreased levels of OPG can lead to excessive activation of RANKL, resulting in an increase in the number of osteoclasts and bone resorption, which contributes to the development of osteoporosis.^[^ ^175^ ^]^ OPG exerts its effects by binding to RANKL, thereby regulating bone remodeling and maintaining balance.^[^ ^176^ ^]^ The role of OPG in the CNS is complex. Ischemia has been shown to upregulate OPG expression,^[^ ^177^ ^]^ and elevated plasma levels of OPG are associated with poor prognosis in ischemic stroke.^[^ ^178^ ^]^ Inhibition of OPG expression has been linked to, suggesting that increased OPG may contribute to decreased RANKL/RANK signaling and heightened post‐ischemic inflammation.^[^ ^177^ ^]^ Conversely, OPG signaling may exert neuroprotective effects in multiple sclerosis by reducing inflammation through the downregulation of RANKL/RANK activity.^[^ ^179^ ^]^ The interplay between neurons and glial cells further complicates the role of OPG in the CNS. Neuron‐derived OPG has been shown to inhibit microglial recruitment and reduce synaptic loss following TBI.^[^ ^180^ ^]^ Although we have a preliminary understanding of the function of OPG in both bone and brain, further research is needed to elucidate its specific mechanisms and roles in disease states. Such studies may provide new insights and potential therapeutic targets for bone‐related disorders and neurodegenerative diseases.

#### 3.2.7 RANKL

RANKL is a key regulator in the formation of osteoclasts. It promotes the maturation, activation, and survival of osteoclasts by binding to its receptor RANK, thereby enhancing bone resorption. The overexpression of RANKL, coupled with reduced levels of OPG, leads to increased osteoclast activity and bone remodeling, ultimately resulting in the development of osteoporosis.^[^ ^175^ ^]^ Both RANKL and RANK are also expressed in the CNS. The RANKL/RANK system in the CNS plays a vital role in regulating body temperature,^[^ ^181^ ^]^ energy metabolism,^[^ ^182^ ^]^ cerebral ischemia,^[^ ^177^ ^]^ and autoimmune encephalitis.^[^ ^179^ ^]^ RANKL activation is involved in the brain regions responsible for thermoregulation, inducing fever via the cyclooxygenase‐2 (COX‐2)/ prostaglandin E2 (PGE2)/ prostaglandin E receptor 3 (EP3R) pathway. Female mice with a deletion of the RANK gene in neurons and astrocytes exhibit elevated baseline body temperatures.^[^ ^181^, ^183^ ^]^ During the acute phase, RANKL and RANK mRNA levels increase and are expressed in activated microglia and macrophages.^[^ ^184^ ^]^ RANKL/RANK signaling can mitigate inflammation through the Toll‐like receptor signaling pathway in microglia.^[^ ^177^ ^]^ Thus, the OPG/RANKL/RANK axis holds promising potential for the treatment of ischemic brain inflammation. Mice with T cell‐specific RANKL deficiency show resistance to experimental autoimmune encephalomyelitis (EAE). Pharmacological inhibition of RANKL can prevent the development of EAE without affecting peripheral immune responses,^[^ ^179^ ^]^ indicating that RANKL could serve as a potential therapeutic target for CNS autoimmune diseases. The interplay of RANKL in bone and brain is complex, and additional research is required to clarify the specific mechanisms by which RANKL operates in various physiological and pathological contexts.

### 3.3 Fractures Exacerbate the Progression and Prognosis of TBI

Notably, unlike the positive effects of TBI on the regulation of fracture healing, the occurrence of fractures worsens TBI.^[^ ^185^ ^]^ Elevated proinflammatory cytokines after TBI were associated with neurological disease and mortality.^[^ ^186^ ^]^ Activation of astrocytes^[^ ^187^ ^]^ and microglia^[^ ^188^ ^]^ worsened the neuroinflammatory response with the release of neurotoxic molecules. It was found that TBI mice combined with fractures had greater encephalopathy, more severe brain oedema.^[^ ^185^ ^]^ High mobility group box 1 (HMGB1) levels were upregulated in fractured mice, which activates a systemic immune response that causes secondary damage to TBI.^[^ ^25^ ^]^ Neutralizing HMGB1 attenuated the infiltration of macrophages and microglia, thereby alleviating the lesions and behavioral dysfunction of TBI associated with fractures.^[^ ^189^ ^]^ Leukocyte‐mediated cerebral inflammation and swelling in TBI with fracture impaired subsequent neurologic recovery, including spatial learning ability and memory.^[^ ^190^, ^191^ ^]^ Interventions that modulate the excessive enhancement of microglia activation and inflammatory response early after TBI may be an effective direction to accelerate neurological recovery and reduce the risk and other adverse complications of TBI.

The phenomenon of fractures exacerbating TBI results from a multifactorial interplay involving mechanisms such as inflammatory responses, hemodynamic changes, and metabolic disturbances. Understanding these mechanisms can assist clinicians in developing more effective treatment strategies for patients with combined injuries, ultimately improving their prognosis. Future research should explore this area to uncover more specific mechanisms and develop targeted interventions.

## 4 Pharmacological Management and Bone Health Risks at Different Stages of TBI

TBI affects bone homeostasis by influencing systemic changes in the immune, endocrine, and sympathetic nervous systems, and these changes are long‐term. In the management of TBI, the selection of pharmacological agents during the acute and recovery phases is crucial for mitigating damage, controlling inflammation, promoting neural repair, and enhancing functional outcomes. Of the medications that have been shown to be effective and have a documented safety profile for use in patients with acute and post‐acute TBI, some may potentially prevent or mitigate osteoporosis, however, there are still a number of medications that is associated with an increased risk of osteoporosis and fracture at the same time.

### 4.1 Acute Phases in TBI

Tranexamic Acid is widely used in emergency and elective surgery to control bleeding, and treatment with Tranexamic Acid within 3 h of TBI may reduce the risk of death in patients with mild to moderate TBI.^[^ ^192^ ^]^ Long‐term Tranexamic Acid stimulation increases osteoblast proliferation and matrix mineralization while inhibiting osteoclastogenesis.^[^ ^193^ ^]^ In addition, systemic Tranexamic Acid administration accelerated early bone formation and fracture healing in rats.^[^ ^194^ ^]^

Mannitol is commonly used to treat increased intracranial pressure and cerebral oedema. Dietary mannitol increased calcium and magnesium absorption in rats.^[^ ^195^ ^]^ Treatment of patients with multiple metacarpal fractures using a combination of steroids and mannitol promotes reduction of hand oedema and pain in the acute postoperative period. Prompt physiotherapy can improve joint mobility and lead to a faster achievement of gripping ability.^[^ ^196^ ^]^

Heparin and oral anticoagulants are commonly used to prevent venous thromboembolism in patients with TBI and fractures after surgery. Long‐term use of antithrombotic medications has a negative impact on bone. Heparin decreases BMD and increases the risk of fragility fractures, so heparin should be used with caution in patients with a history of osteoporosis and in elderly patients.^[^ ^197^ ^]^ Low molecular weight heparin (LMWH) appears to be safer than heparin. The new direct oral anticoagulants have been found to be safe for bone health.^[^ ^198^ ^]^

Methylprednisolone improves neurological function and reduces secondary injury following TBI. Low doses of methylprednisolone can decrease bone resorption and exhibit bone‐protective effects.^[^ ^199^ ^]^ However, serum alkaline phosphatase and osteocalcin levels decrease at higher doses, and there is a significant reduction in the number of osteoclasts and osteoblasts in the tibial metaphysis.^[^ ^200^ ^]^ The method of administration of methylprednisolone also has varying impacts. In patients with rheumatoid arthritis receiving oral methylprednisolone, significant reductions in BMD at the lumbar spine, femoral neck, and total body were observed at 6 and 12 months. In contrast, high‐dose methylprednisolone pulse therapy did not result in significant reductions in BMD at any site.^[^ ^201^ ^]^

Phenytoin is an anticonvulsant medication that prevents seizures in TBI. It stabilizes neuronal membranes, inhibits the release of excitatory neurotransmitters, and potentially mitigates secondary brain injury.^[^ ^202^ ^]^ Additionally, phenytoin regulates the osteogenic differentiation of human mandibular BMSCs via the PI3K/Akt signaling pathway.^[^ ^203^ ^]^ However, long‐term administration of phenytoin has been associated with bone loss, which may be attributable to deficiencies in micronutrients.^[^ ^204^ ^]^

### 4.2 Recovery Phases in TBI

Various selective serotonin reuptake inhibitors (SSRIs) are utilized to ameliorate symptoms of depression and anxiety, thereby facilitating emotional recovery. Sertraline, citalopram, and fluoxetine have been shown to be beneficial in treating post‐TBI depression.^[^ ^205^ ^]^ However, sertraline has been reported to adversely affect bone healing by impairing bone repair and regeneration,^[^ ^206^ ^]^ with a significant reduction in bone formation observed in bone defects in rat models.^[^ ^207^ ^]^ Furthermore, exposure to citalopram and sertraline has been found to compromise embryonic bone development.^[^ ^208^ ^]^ Long‐term use of fluoxetine may disrupt sphingolipid metabolism in bone marrow adipose tissue, consequently accelerating bone loss.^[^ ^209^ ^]^ In summary, while SSRIs demonstrate considerable efficacy in the treatment of depression and anxiety, their potential inhibitory effects on bone generation warrant attention, particularly in patients requiring prolonged administration of these medications. Clinicians should regularly assess patients' bone health during treatment and implement appropriate preventive measures as necessary.

Baclofen and tizanidine are commonly utilized to alleviate muscle spasms associated with TBI and other neurological disorders, while also providing beneficial effects in pain relief and improving the sleep quality of patients.^[^ ^205^ ^]^ However, their potential impacts on bone health should not be overlooked. Long‐term intrathecal administration of baclofen may have detrimental effects on bone mineral content, BMD, and body composition.^[^ ^210^ ^]^ Studies have indicated that long‐term use of baclofen and tizanidine may increase the risk of osteoporosis.^[^ ^211^ ^]^ Patients receiving prolonged treatment with these medications should undergo regular assessments of bone health to prevent osteoporosis and related fracture risks.

In the management of TBI, pharmacological treatment plays a crucial role. The aforementioned list includes several commonly used medications that may simultaneously affect bone development during the treatment of TBI. While these medications contribute positively to the improvement of neurological functions, reduction of inflammation, and promotion of neural repair, they may also adversely affect bone metabolism, leading to increased risks of osteoporosis and fractures. Currently, several drugs have demonstrated good safety and efficacy profiles in the acute and recovery phases of TBI management. However, a significant number of medications remain inadequately studied, particularly regarding their potential impact on bone health. Future research efforts must delve deeper into this area to enhance the understanding of how these drugs affect bone development and to clarify the suitability and safety of various medications in patients with TBI. Moreover, the findings from these studies provide critical guidance for the clinical treatment of patients with TBI. This is particularly important for elderly patients or those with pre‐existing osteoporosis, where the selection of medications should be approached with greater caution. Clinicians must not only focus on the therapeutic effects of drugs on TBI itself but also consider their possible side effects, especially concerning bone metabolism. For instance, certain commonly used antiepileptic drugs and corticosteroids, while essential for controlling seizures and inflammatory responses in patients with TBI, may also lead to decreased bone density, thus heightening the risk of fractures. Therefore, when developing treatment plans for elderly patients with TBI, clinicians should consider the overall health status of the patient, their bone health, and the potential side effects of the medications prescribed, to achieve the best possible therapeutic outcomes.

## 5 Strategies for Maintaining the Balance of Brain–Bone Axis

### 5.1 Interactions Between Bone and TBI

TBI is a complex pathological condition that not only causes significant damage to the nervous system but also has a considerable impact on the skeletal system. TBI impacts bone metabolism and increases the risk of osteoporosis as previously described. Therefore, prevention and management of bone diseases such as osteoporosis and osteoarthritis are crucial. Focus on risk factors for osteoporosis in patients with TBI and intervene early to prevent it. Early detection of symptoms such as osteoporosis and fractures in patients with TBI allows assessment of the risk and severity of patients to take appropriate medical measures and treatment plans. It has been found that braking after TBI induces low bone mass and the development of osteoporosis.^[^ ^31^ ^]^ Sedentary behavior and BMD were negatively correlated.^[^ ^212^ ^]^ Physical activity and exercise could increase BMD and improve bone strength in patients with osteoporosis.^[^ ^213^ ^]^ Therefore, moderate exercise and movement can promote recovery from TBI and prevent osteoporosis. Exercise may also reduce the likelihood of the occurrence of cognitive impairment, among other things, in patients with TBI.^[^ ^214^ ^]^ Overall, exercise may be the most cost‐effective and accessible strategy for alleviating TBI and preventing osteoporosis. TBI promotes exacerbated callus formation and accelerates fracture healing. Bone healing and repair is a complex and dynamic biological process, and the role of the CNS in it lacks more detailed study. Hence, elucidating the mechanism of accelerated osteogenesis in TBI will not only provide cogent evidence for neuromodulation of bone, but also suggest potential therapies for treating delayed bone healing or bone nonunion.

**Figure 5:**
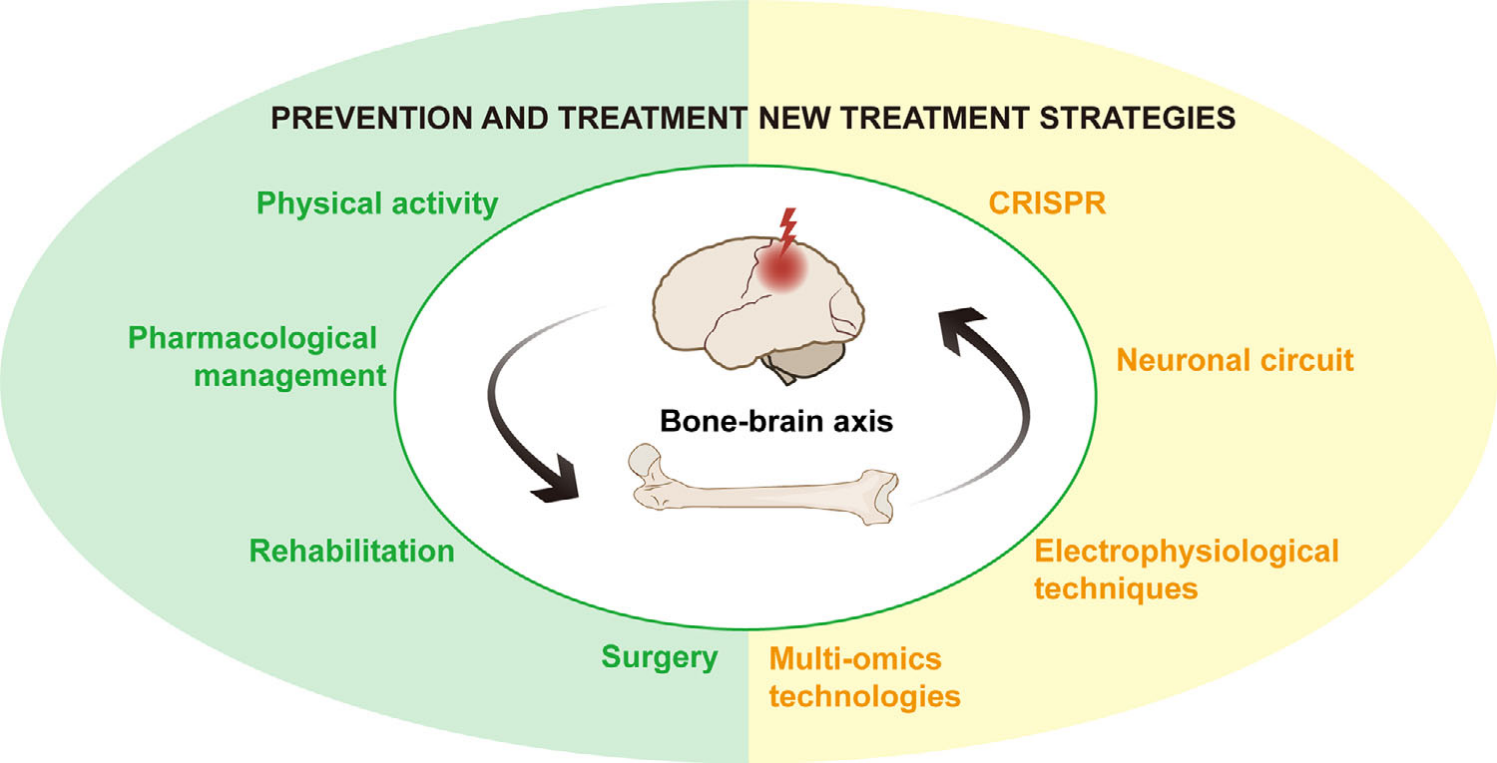
Insights for prevention and treatment strategies based on brain–bone axis. The figure illustrates the significance of research on the brain–bone axis in the prevention and treatment of diseases such as TBI and osteoporosis. Various interventions, including physical activity, pharmacological management, rehabilitation, and surgical approaches, are investigated to effectively mitigate the onset and progression of these diseases. The rapid advancement of emerging technologies such as CRISPR, neural circuit techniques, electrophysiological methods, and multiomics approaches is ushering the study of the brain–bone axis into a new era. These technologies not only enhance experimental precision but also provide novel insights into the complex interactions between the nervous and skeletal systems. Such advancements are expected to facilitate the development of more effective therapeutic strategies, thereby improving the prognosis for related diseases.

Bone‐related factors, such as BMPs, as well as BMSCs, play different roles following TBI. These factors directly or indirectly influence bone regeneration and repair, as well as the prognosis of brain injuries, while BMSCs are crucial for local neuroprotection and regeneration. At the same time, the interplay between fractures and TBI is significant. Research has shown that fractures may exacerbate the pathological processes of brain injuries, leading to more severe neurological deficits and poorer outcomes. This complex interaction highlights the intricate crosstalk between the brain and the skeletal system (**Figure** **5**). Exploring the underlying mechanisms in greater depth could provide new insights and therapeutic approaches for clinical intervention.

### 5.2 Management and Application of the Brain–Bone Axis

The brain–bone axis has garnered increasing attention in recent years, highlighting its significance in elucidating the interactions between the CNS and the skeletal system, as well as their implications for both physiological and pathological states. This nexus not only encompasses the regulation of bone metabolism but also underscores the critical role of the nervous system in maintaining skeletal health.

#### 5.2.1 Prevention of Osteoporosis and Treatment of Fracture Healing

Dysregulation of the hypothalamic–pituitary axis following TBI disrupts the systemic regulation of bone homeostasis, leading to the inevitable occurrence of osteoporosis. Stimulation of glutamatergic neurons from SFO to PVN can promote the upregulation of serum PTH and facilitate the restoration of bone mass.^[^ ^14^ ^]^ PTH_1‐34_ has been shown to increase trabecular bone volume and promote bone formation, while also inhibiting the senescence of astrocytes and the activation of pro‐inflammatory cytokines in the brain.^[^ ^21^ ^]^ This suggests the potential of PTH as a therapeutic agent for mitigating osteoporosis induced by TBI, highlighting its role as a promising treatment option for both conditions.

Following TBI, the sEVs enriched with miR‐328a‐3p and miR‐150‐5p can target the FOXO4 and CBL 3′‐UTR, thereby promoting osteogenesis. Moreover, hydrogels loaded with sEVs containing miR‐328a‐3p have been shown to significantly accelerate bone healing and repair cranial defects.^[^ ^132^ ^]^ This provides critical evidence and research directions regarding the role of central regulation in bone formation, establishing a clear link between injured neurons and osteogenic therapeutics in both animal models and clinical settings.

BMP‐2, FGF‐2, IL‐1β, and PDGF are upregulated in the serum of patients with femoral fractures complicated by TBI, indicating their involvement in bone healing.^[^ ^135^ ^]^ In mice with fractures and TBI, the number of mast cells and the expression of CXCL10 are significantly reduced.^[^ ^122^ ^]^ Furthermore, SDF‐1 recruits mesenchymal stem cells that participate in endochondral bone repair under TBI.^[^ ^137^ ^]^ Additionally, CB1‐mediated osteogenic metabolic responses have been found to play a role in the healing of cranial defects following mild TBI.^[^ ^215^ ^]^ These findings suggest that further in‐depth and effective research is warranted.

#### 5.2.2 Treatment and Prognosis of TBI and Neurodegenerative Diseases

Bone‐derived proteins may represent a novel potential therapeutic approach targeting the brain. OCN, SOST, and BMP are secreted by osteoblasts and osteocytes and are responsible for regulating bone homeostasis. OCN has been validated as a neuroprotective and cognition‐enhancing peptide.^[^ ^216^ ^]^ Therefore, in‐depth exploration of both basic and clinical research focused on OCN as a treatment for TBI and other diseases holds significant potential. Reducing bone‐derived SOST may mitigate synaptic damage,^[^ ^170^ ^]^ however, research investigating its therapeutic implications for brain injury remains limited. Other bone‐derived proteins exhibit similar constraints. Nevertheless, from the perspective of the bone–brain axis, there is a pressing need for future investigations to further explore the potential of bone‐derived protective factors and monoclonal antibodies in the treatment of TBI through precise targeting.

BMSCs have emerged as a promising regenerative therapeutic strategy due to their multipotent differentiation capabilities, immunomodulatory properties, and neuroprotective effects. Phase II clinical trials have demonstrated that intravenous infusion of autologous BMSCs is safe and feasible in treating adult patients with severe TBI, effectively reducing critical inflammatory biomarkers.^[^ ^141^ ^]^ This suggests that BMSCs transplantation for TBI is a promising and predictable therapeutic approach. EVs derived from BMSCs possess significant potential in therapeutic applications. Administration of BMSC‐derived EVs has been shown to alleviate microglial activation and the activation of inflammatory factors.^[^ ^150^ ^]^ Intra‐ventricular delivery of these EVs has improved behavioral cognitive deficits in AD mice while inhibiting the overactivation of microglia and astrocytes in the hippocampus, simultaneously promoting the upregulation of synaptic‐related proteins and BDNF.^[^ ^151^ ^]^ Moreover, the intranasal delivery of EVs is considered safe and less invasive, making it a potential therapeutic approach for TBI.^[^ ^217^ ^]^ Nevertheless, research on the use of BMSCs‐derived EVs for treating TBI and neurodegenerative diseases is still in its infancy, with a lack of comprehensive clinical trials. The feasibility, safety, and efficacy of their clinical applications require further exploration.

Understanding the underlying mechanisms by which the brain influences bone health could pave the way for innovative therapeutic strategies. For instance, targeting specific pathways within the brain–bone axis may offer novel approaches to mitigate bone loss associated with aging or injury, thereby enhancing the quality of life for elderly populations and individuals recovering from fractures. Moreover, the exploration of the brain–bone axis holds promise for clinical applications. By modulating neurological functions, it may be possible to improve bone health outcomes, providing more effective interventions for at‐risk groups. This could lead to the development of new treatment modalities that focus on the integration of neurological and orthopedic care, thereby addressing the multifaceted nature of conditions affecting both systems.

As research in this domain advances, findings related to the brain–bone axis have the potential to reshape our understanding of the intricate relationship between the nervous and skeletal systems. This evolving perspective may not only challenge traditional concepts but also open new avenues for biomedical research, ultimately leading to enhanced therapeutic interventions and improved patient outcomes. Future studies will be essential to unravel the complexities of this axis, offering insights that could transform clinical practices and enhance our ability to address bone‐related health issues in diverse populations.

### 5.3 Application of New Treatment Strategies

The development and application of technologies in preclinical research have defined the mechanisms that can be targeted and provided new therapeutic strategies for bone disease and TBI. Emerging new biological technologies can deepen or overturn existing research and feed back to basic neuroscience and orthopedics. The use of gene knockout mouse technology, particularly through gene editing techniques like CRISPR‐Cas9,^[^ ^218^ ^]^ allows researchers to inactivate specific genes and study their functions within an organism. By knocking out genes related to bone metabolism or neuronal signaling, we can investigate their specific roles in the brain–bone axis and how they influence the communications between the brain and bone. Furthermore, applying specific disease models, such as osteoporosis and neurodegenerative diseases, to these knockout mice enables a more direct observation of how these conditions impact the brain–bone axis.

Neuronal circuit techniques, including optogenetics and chemogenetics, allow for precise control over specific populations of neurons. Neuronal projections and neural circuit function can be realized with the techniques of optogenetics and chemical genetics.^[^ ^14^, ^20^, ^219^ ^]^ By activating or inhibiting particular neural circuits and combining this with behavioral experiments, we can explore the roles of these circuits in regulating bone health and metabolism. Additionally, integrating electrophysiological techniques to record neuronal electrical activity provides real‐time monitoring of the nervous system's functional state, revealing the electrical signal interactions between the nervous system and bone. To elaborate further, functional imaging techniques^[^ ^220^ ^]^ may help decipher the link between the nervous system and bone homeostasis. Transcranial direct current stimulation (tDCS) and transcranial magnetic stimulation (TMS) can activate or inhibit neuronal activity by applying a low‐intensity electrical current in specific regions of the cerebral cortex. These may be a key research focus for future clinical treatment directions. Combining single‐cell and spatial transcriptomics can identify and accurately localize functionally relevant transcripts,^[^ ^221^ ^]^ significantly enhancing our understanding of cellular heterogeneity and microenvironments. This approach aids in identifying cell populations that play critical roles in the interactions between the brain and bone, thus shedding light on the spatial dynamics of the brain–bone axis.

With the advancement of emerging technologies such as gene knockout mice, neuronal circuit techniques, electrophysiological methods, and multiomics technologies (Figure 5), research on the brain–bone axis is entering a new phase. These technologies not only provide more precise experimental tools but also offer new perspectives for understanding the interactions between the nervous system and the skeletal system. In the future, the integration of these techniques will foster interdisciplinary research, revealing complex biological mechanisms, enhancing our understanding of the molecular interplay between the brain and bone, and potentially uncovering new therapeutic targets.

### 5.4 Bridging Disciplines for Enhanced Understanding and Treatment of Brain–Bone Interactions

A comprehensive framework of interdisciplinary thinking, methods, and research strategies is necessary to identify the best translational pathways to accelerate fracture healing and treat TBI. Better visualization of nervous system and bone interactions at multiple scales and levels helps to select the most promising therapies for individual patients in the future. Therefore, targeting the mechanisms underlying brain and bone crosstalk for drug development needs to be performed simultaneously. Since targeting mechanisms of TBI and bone disease are often also associated with normal nervous system and bone development, we can identify how to prevent osteoporosis or treat TBI in the conventional state through studies of pathological conditions. In conclusion, considering the complex crosstalk between brain and bone, identifying key molecules that contribute to osteoporosis or accelerate fracture healing in TBI will be crucial to future therapies.

Future research trends indicate that cross‐organ studies will become a crucial direction for understanding the complex interactions within biological systems, such as the brain–gut–bone axis.^[^ ^222^ ^]^ These investigations not only elucidate the interactions between different physiological systems but also provide new perspectives on the mechanisms of disease and potential treatment strategies. The composition and function of the gut microbiome are believed to have profound effects on the host's metabolism, immune function, and neurological health. By regulating the gut microbiome—through methods such as probiotics or prebiotics—it may be possible to enhance neurological function and promote bone health,^[^ ^223^ ^]^ offering a novel integrated therapeutic approach. Moreover, changes in the gut microbiome could serve as biomarkers for diseases related to the brain–bone axis, including osteoporosis and depression. Interdisciplinary collaboration is key by integrating genomic, transcriptomic, metabolomic, and microbiomic data; researchers can gain a more comprehensive biological perspective that reveals interactions across different systems. Utilizing modeling and computational biology to construct multidimensional biological system models can aid in predicting the effects of various treatment strategies. Most importantly, such collaboration can accelerate the translation of fundamental research findings into clinical applications, creating new possibilities for the integration of basic research and clinical practice, and ultimately providing patients with more comprehensive treatment options.

### 5.5 Insights for Drug Development and Clinical Applications Based on Brain–Bone Axis

Research on the brain–bone axis has garnered significant attention in recent years, particularly in the realms of drug development and clinical applications, providing us with new therapeutic strategies and directions. By delving into the mechanisms underlying the brain–bone axis, researchers can identify key molecules and signaling pathways associated with this interaction, paving the way for the development of targeted drugs. These novel therapies not only aim to improve treatment outcomes for conditions such as TBI and osteoporosis but may also offer new solutions for related diseases.

In studies of the brain–bone axis, several critical molecules and signaling pathways have been found to play a vital role in the interaction between the nervous system and the skeletal system. Neurotrophic factors, such as BDNF^[^ ^224^ ^]^ and neurotrophins,^[^ ^225^ ^]^ are involved in neuroprotection regulation. Hormones related to bone metabolism, including PTH,^[^ ^14^ ^]^ estrogen,^[^ ^226^ ^]^ and insulin,^[^ ^227^ ^]^ are crucial in the regulation of bone homeostasis (**Table** **1**). Additionally, inflammatory factors play an important role in the pathogenesis of diseases such as neural injury and osteoporosis. By conducting in‐depth research on these molecules and developing new targets, we can design drugs with higher selectivity and efficacy.

**Table 1:** Neurohormones, neuropeptides, and neurotransmitters in the crosstalk of brain–bone following TBI.

Neurohormone	Target	Effect and reference
GH	Osteoblast	Induce proliferation in osteoblasts by increasing IGF‐1 production.^[^ ^34^ ^]^
GC	Osteoclast	Enhance bone resorption by promoting osteoclastogenesis through the activation of RANKL.^[^ ^42^ ^]^
	BMSCs	Promote lipogenic differentiation and inhibit bone formation in BMSCs.^[^ ^44^, ^47^ ^]^
ACTH	Osteoblast	Promote osteogenic differentiation and maintain bone mass.^[^ ^52^ ^]^
PTH	Osteocyte	Promote RANKL expression in osteocytes to increase osteoclast number and function.^[^ ^54^ ^]^
	Osteoblast	Intermittent PTH enhances osteoblast survival and osteogenic differentiation.^[^ ^55^ ^]^
Glucagon and insulin	Osteoblast	A low bone turnover.^[^ ^59^ ^]^
	Osteoclast	
FSH	Osteoclast	Enhance osteoclastic differentiation and bone resorption.^[^ ^61^ ^]^
Leptin	Osteoblast	Leptin deficiency retarded the rate of fracture healing.^[^ ^108^, ^109^ ^]^
PRL	Osteoblast	Increase RANKL and decrease OPG expression in osteoblasts.^[^ ^110^ ^]^
**Neuropeptide**	**Target**	**Effect and reference**
NPY	BMSCs	Inhibit osteogenesis and promote adipogenesis in BMSCs.^[^ ^66^ ^]^
CART	Osteoclast	Increase the number of osteoclasts to promote bone resorption.^[^ ^72^, ^73^ ^]^
CGRP	BMSCs	Enhance osteogenic differentiation of BMSCs through the PKA/cAMP response element‐binding protein (CREB)/JUNB pathway to induce the activation of osterix.^[^ ^115^, ^116^, ^117^ ^]^
Substance P	Chondrocyte	SP deficiency delays the terminal differentiation of hypertrophic chondrocytes.^[^ ^121^ ^]^
**Neurotransmitter**	**Target**	**Effect and reference**
NMDA	Osteoblast	Stimulate osteoblasts differentiation through PKA, PKC, PI3K signaling pathways.^[^ ^77^ ^]^
5‐HT	Osteoblast	Lead to higher bone mineral density, affect bone architecture, and lead to higher femoral bone stiffness.^[^ ^81^ ^]^
Dopamine	Osteoclast	Agonists inhibit osteoclast differentiation.^[^ ^84^ ^]^
	BMSCs	Osteogenic mineralization is significantly increased.^[^ ^85^ ^]^
Acetylcholine	Osteoblast	mAChR M3R stimulate cancellous bone microarchitecture, bending stiffness, and bone matrix synthesis.^[^ ^90^, ^91^ ^]^
	Osteoclast	Osteoclastogenesis is reduced in α7 KO mice.^[^ ^17^ ^]^
Norepinephrine	Osteoblast	MicroRNA‐21 is upregulated in osteoblasts due to the activation of βARs and stimulates osteoclasts via exosomal transport to induce osteoporosis.^[^ ^95^ ^]^
	Periosteal cells	Stimulate the expression of VEGF and CGRP in periosteal cells via ADRB2 and thereby promote osteogenic H‐vessel formation in fracture scab after TBI.^[^ ^124^ ^]^

The bioavailability and targeting of drugs are significant factors affecting treatment outcomes. Innovations in drug formulation, such as nanoparticle carriers and controlled‐release systems,^[^ ^228^ ^]^ have become key directions for enhancing therapeutic effectiveness. Utilizing polymer‐based nanoparticles to encapsulate drugs can improve their solubility and stability while enabling targeted release. Hydrogels,^[^ ^229^ ^]^ as a novel controlled‐release carrier, can release drugs under specific conditions, facilitating advancements in tissue engineering and regenerative medicine. The study of the brain–bone axis holds substantial significance not only in basic scientific research but also in clinical applications, with the potential for far‐reaching impacts.

In summary, research on the brain–bone axis provides new perspectives and opportunities for drug target development and clinical application. By identifying key molecules and signaling pathways, innovating drug formulations, and leveraging advancements in materials science, we can develop more effective treatment approaches to improve outcomes for conditions such as TBI and osteoporosis. These studies not only aim to enhance patients' quality of life but also have the potential to open new avenues for future biomedical research.

## 6 Conclusion

In this review, we thoroughly explore the interplay between TBI and bone, extending the discussion to the significance of the brain–bone axis. Based on experimental evidence gathered from physiological and pathological processes, we reveal the structural and functional interactions between the nervous and skeletal systems. The concept of the brain–bone axis and its in‐depth study may yield further insights and guidance applicable to neurological and skeletal disorders to develop safe and effective treatment options.

## Conflict of Interest

The authors declare no conflict of interest.

## Author Contributions

Z.J. gave a brief introduction to this article. Z.W. was responsible for manuscript writing. Z.W. drew figures. Z.L.L. revised the manuscript. All authors approved the final version of this manuscript.
